# Antioxidant activity of *Coriandrum sativum* and protection against DNA damage and cancer cell migration

**DOI:** 10.1186/1472-6882-13-347

**Published:** 2013-12-09

**Authors:** Esther LH Tang, Jayakumar Rajarajeswaran, Shin Yee Fung, MS Kanthimathi

**Affiliations:** 1 Department of Molecular Medicine, UMCPR (University of Malaya Centre for Proteomics Research), Faculty of Medicine, University of Malaya, 50603 Kuala Lumpur, Malaysia

**Keywords:** *Coriandrum sativum*, Antioxidants, Anticancer activity, MCF-7, Hydrogen peroxide, Caspase, Cell cycle, DNA damage, Cancer cell migration, Comet assay

## Abstract

**Background:**

*Coriandrum sativum* is a popular culinary and medicinal herb of the Apiaceae family. Health promoting properties of this herb have been reported in pharmacognostical, phytochemical and pharmacological studies*.* However, studies on *C. sativum* have always focused on the aerial parts of the herb and scientific investigation on the root is limited. The aim of this research was to investigate the antioxidant and anticancer activities of *C. sativum* root, leaf and stem, including its effect on cancer cell migration, and its protection against DNA damage, with special focus on the roots.

**Methods:**

Powdered roots, leaves and stems of *C. sativum* were extracted through sequential extraction using hexane, dichloromethane, ethyl acetate, methanol and water. Total phenolic content, FRAP and DPPH radical scavenging activities were measured. Anti-proliferative activitiy on the breast cancer cell line, MCF-7, was assayed using the MTT assay. Activities of the antioxidant enzymes, catalase, superoxide dismutase, glutathione peroxidase, and of the caspases-3, -8 and -9 were assayed on treatment with the extract. Cell cycle progression was analysed using flow cytometry. The scratch motility assay was used to assess inhibition of MCF-7 cell migration. DNA damage in 3 T3-L1 fibroblasts was evaluated by the comet assay. The components in the extract were identified by HPLC and GC-MS.

**Results:**

The ethyl acetate extract of C*. sativum* roots showed the highest antiproliferative activity on MCF-7 cells (IC_50_ = 200.0 ± 2.6 μg/mL) and had the highest phenolic content, FRAP and DPPH scavenging activities among the extracts. *C. sativum* root inhibited DNA damage and prevented MCF-7 cell migration induced by H_2_O_2_, suggesting its potential in cancer prevention and inhibition of metastasis. The extract exhibited anticancer activity in MCF-7 cells by affecting antioxidant enzymes possibly leading to H_2_O_2_ accumulation, cell cycle arrest at the G_2_/M phase and apoptotic cell death by the death receptor and mitochondrial apoptotic pathways.

**Conclusions:**

This study is the first report on the antioxidant and anticancer properties of *C. sativum* root. The herb shows potential in preventing oxidative stress-related diseases and would be useful as supplements used in combination with conventional drugs to enhance the treatment of diseases such as cancer.

## Background

Plants have played a significant role in providing the human race with remedies. At present, phytotherapy is a recognised complementary and alternative medicinal (CAM) therapeutic modality. It is one of the promising fields in health care as supportive medicine in the treatment of diseases like cancer [1].

Reactive oxygen species (ROS), such as hydroxyl radicals and hydrogen peroxide (H_2_O_2_), are constantly generated as normal by-products of mitochondrial respiration. An imbalance between the generation of ROS and cellular antioxidant capacity can lead to oxidative stress. Among the pathologies linked to oxidative stress are atherosclerosis, hypertension, diabetes, inflammation, Parkinson’s and Alzheimer’s diseases, and cancer [2]. The human body has innate defense mechanisms to counter ROS in the form of antioxidant enzymes such as superoxide dismutase (SOD), glutathione peroxidase (GPx) and catalase (CAT). Consumption of dietary plants as food containing phytochemicals with antioxidant properties, such as phenolics and ascorbic acid, can help strengthen the antioxidant balance of the body [3].

H_2_O_2_ is an oxidizing agent that can be converted to reactive hydroxyl radicals and has been associated with DNA damage, mutations and genetic instability, which can lead to cancer development [4]. H_2_O_2_ can increase cancer cell proliferation and migration [5] resulting in metastasis, which is the leading cause of cancer deaths and ineffectiveness of chemotherapeutic drugs. Plant bioactives may act as cancer chemopreventive agents in normal cells by keeping H_2_O_2_ levels within physiological levels, thus preventing DNA damage. At the same time, plant extracts can also act as chemotherapeutic agents by increasing H_2_O_2_ in rapidly dividing cancer cells to levels that cannot be counterbalanced by the cellular antioxidant systems, producing apoptotic cell death [6]. Natural products have been recognized as inducers of apoptosis in tumor cells of human origin [7]. Consequently, considerable attention has been focused towards natural products as the source of new chemotherapeutic and chemopreventive agents.

*Coriandrum sativum L.* is a culinary and medicinal herb of the Apiaceae family commonly known as coriander. The roots and leaves of *C. sativum* are rich with aromatic flavour and are popularly used in soups in Thai and other Asian cooking. Traditionally, the plant parts are used to alleviate spasms, gastric complaints, bronchitis, gout and giddiness [8]. Previous studies on this herb show their various medicinal properties, including antidiabetic, antioxidant, hypocholesterolemic, antihelmintic, antibacterial, hepatoprotective, anticancer and anxiolytic activities [9,10]. The phenolic compounds, apigenin, catechin and *p-*coumaric acid, and aliphatic alkenals and alkanals were reported in *C. sativum* aerial parts [11,12] while linalool, geranyl acetate and petroselinic acid were found in the fruit [13]. Reviews on *C. sativum* reported various novel pharmacognostical, phytochemical and pharmacological studies carried out on the plant [9,10]. However, studies on *C. sativum* have always focused on the aerial parts of the herb [14,15]. Investigations on the medicinal properties of *C. sativum* roots in scientific literature are scanty and limited, to virtually none. Yet, the roots of this plant are often used in cooking and traditional medicine and are thought to contribute to health and protection against the onset of disease. Therefore, it is imperative that studies should be conducted to investigate the unexploited potential of *C. sativum* roots. The vast health promoting properties associated with the intake of *C. sativum* in the diet further warrant the herb for study. The main aim of this work was to investigate the antioxidant and anticancer activities of *C. sativum* and its protection against DNA damage in normal cells and MCF-7 cell migration induced by H_2_O_2_*in vitro*. This is the first study reporting the antioxidant and anticancer effects of *C. sativum* root extract on the breast cancer cell line, MCF-7.

## Methods

### Chemicals

Analytical grade chemicals were purchased from Fisher Scientific (UK) and Merck (Germany). Dimethyl sulfoxide (DMSO) and H_2_O_2_ were purchased from Univar (Australia). High performance liquid chromatography (HPLC) grade chemicals and standards, gallic acid, quercetin, rutin, colchicine and mitomycin C were obtained from Sigma Chemical Co. (UK). HPLC grade acetonitrile was purchased from F S Chemicals (India). Ultrapure water used was purified using the Milli-Q-plus filter system by Millipore (USA).

### Plant material

Fresh *Coriandrum sativum* roots, leaves and stems were purchased from the wet market in Selayang, Kuala Lumpur, Malaysia. The plant was identified by Dr. M. Sugumaran, Institute of Biological Sciences, University of Malaya. A voucher specimen (KLU47742) was deposited in the University of Malaya Herbarium. *C. sativum* roots were separated from the leaves and stems using a knife. The plant parts were washed under running tap water to remove dirt and soil and finally rinsed with distilled water. The plant parts were freeze-dried, weighed, ground into fine powder and stored at -20°C until extraction.

### Preparation of plant extracts

Powdered roots, leaves and stems of *C. sativum* were extracted through sequential extraction using hexane, dichloromethane, ethyl acetate, methanol and water. Briefly, powdered roots (20 g) and powdered leaves and stems (120 g) were extracted in 100 and 600 ml of hexane (1:5 w/v), respectively, for 6 h at 40°C on a hotplate with stirring. Extracts were then filtered through filter paper Whatman no. 1 and the resulting plant residues were re-extracted twice with fresh hexane. The remaining plant residue was then extracted using dichloromethane, followed by ethyl acetate, methanol and water (three times in each solvent). Each filtrate (except for the aqueous extract) was concentrated to dryness under reduced pressure at 40°C using a rotary evaporator. The aqueous extract was concentrated to dryness in a freeze-dryer. The dried extracts were stored at -20°C.

For use in cell culture treatment, the dried extracts were dissolved in DMSO and diluted in ultrapure water to obtain stock solutions which were sterile filtered through 0.2 μm syringe filters. Stock solutions were diluted in ultrapure water to make appropriate extract concentrations for testing. The final concentration of DMSO in the cell culture reaction mixture was less than 1%. Extracts were kept at 4°C.

### Determination of total phenolic content

Total phenolic content (TPC) of *C. sativum* extracts was determined using the Folin-Ciocalteau method [16] with some modifications. Briefly, 500 μl of 1:10 Folin-Ciocalteau phenol reagent was added to 10 μl of sample (dissolved in 10% DMSO), standard or positive control. The mixture was allowed to stand for 5 min before the addition of 350 μl of 10% sodium carbonate (Na_2_CO_3_). The resulting reaction mixture was incubated in the dark at room temperature (RT) for a further 2 h. Absorbance was then measured at 765 nm using a spectrophotometer. Gallic acid (50–500 mg/l in 10% DMSO) was used as the standard. Rutin and quercetin were used as positive controls. Results were expressed in milligrams of gallic acid equivalents (GAE) per gram dried extract. All experiments were carried out in triplicate.

### Ferric reducing antioxidant power (FRAP) assay

The antioxidant activity based on the ferric reducing ability of *C. sativum* extracts was estimated based on the assay by Benzie & Strain [17] with some modifications. A working reagent was prepared fresh by mixing 10 ml of 300 mM acetate buffer with 1 ml of 10 mM 2,4,6-tripyridyl-s-triazine (TPTZ) in 40 mM of hydrochloric acid (HCl) and 1 ml of 20 mM FeCl_3_.6H_2_O. The freshly prepared FRAP reagent was pre-warmed at 37°C for 5 min after which a blank reading was taken at 595 nm using a plate reader. Subsequently, 3 μl of sample, standard or positive control (each dissolved in 10% DMSO) and 9 μl of water was added to 90 μl of the FRAP reagent. Absorbance readings were measured instantly upon addition of the FRAP reagent and again at 4 min after the start of the reaction. The change in absorbance in the 4 min reaction was calculated by comparison to the absorbance changes of FeSO_4_.7H_2_O against a standard curve (100–1000 μM) tested in parallel. Rutin and quercetin were used as positive controls. Results were expressed as mmol ferric reducing activity of the extracts per gram of dried extract. All experiments were carried out in triplicate.

### DPPH radical scavenging activity

Radical scavenging activities of *C. sativum* sequential extracts were determined by 1,1-diphenyl-2-picrylhydrazyl (DPPH) radical scavenging assay [18] with some modifications. The extract (20 μl) was added to 120 μl of 0.04 mg/ml DPPH solution in methanol. The extracts tested ranged from 0–5000 μg/ml (dissolved in 10% DMSO). The mixtures were mixed well and incubated in the dark for 30 min. The reduction of DPPH absorption was measured at 515 nm using a plate reader. Rutin and quercetin were used as the positive controls. All determinations were performed in triplicate. The DPPH radical scavenging activity was calculated using the following equation:

$$\begin{array}{ll} \mathrm{Percentage}\;\mathrm{inhibition}= & \left(\mathrm{Absorbanc}e_{\mathrm{control}}-\mathrm{Absorbanc}e_{\mathrm{sample}}\right)\\ & \;/\mathrm{Absorbanc}e_{\mathrm{control}}\times 100 \end{array}$$

The IC_50_ value is the concentration of the plant extract required to scavenge 50% of the total DPPH radicals available.

### Cell lines and cell culture

The human breast adenocarcinoma cell line, MCF-7, and the human mammary epithelial cell line, 184B5, were used in the anti-proliferation study. Assays of antioxidant enzymes and caspase activities, cell cycle analysis, and inhibition of cell migration were performed using MCF-7 cells. Mouse fibroblast cells, 3 T3-L1, were used in the comet assay. All cells were purchased from the American Type Culture Collection (ATCC), USA. MCF-7 cells were routinely cultured in RPMI-1640 (Sigma, UK). 3 T3-L1 cells were grown in Dulbecco’s modified Eagle’s medium (DMEM) (Lonza, USA). 184B5 cells were cultured in Mammary Epithelial Basal Medium (MEBM) and supplemented with bovine pituitary extract (BPE), hydrocortisone, human epidermal growth factor (hEGF) and insulin using Mammary Epithelial Cell Growth Medium (MEGM) SingleQuots from Lonza, USA. All cells were supplemented with 10% (v/v) fetal bovine serum (FBS), 100 IU/ml penicillin and 100 μg/ml streptomycin. Cells were grown at 37°C in a humidified incubator with 5% CO_2_.

### Anti-proliferative activity

The inhibition of MCF-7 cell proliferation by *C. sativum* extracts was estimated using the MTT (3-(4,5-dimethylthiazol-2-yl)-2,5-diphenyltetrazolium) bromide assay as described by Mosmann [19]. The ethyl acetate extract of *C. sativum* root showed the best antiproliferative activity on MCF-7 and was assessed for its toxicity on the human mammary cell line, 184B5. Briefly, cells supplemented with 5% FBS were seeded (5 × 10^3^ cells/well) in 96-well microtiter plates and cultured at 37°C in a humidified atmosphere of 5% CO_2_. After 24 h of incubation, the cells were treated with various concentrations of extract (0–500 μg/ml) for another 48 h. Vehicle-control wells with cells only and diluent-control wells with similar DMSO concentrations as in treatment were included. At the end of the incubation period, 10 μl of 5 mg/ml MTT bromide in phosphate-buffered saline (PBS) was added to each well. The plates were re-incubated for a further 4 h after which media and MTT were removed by aspiration. DMSO (100 μl) was added to each well to dissolve the formazan crystals. Absorbance was read using a microtiter plate reader at 595 nm. All measurements were performed in triplicate. The percentage inhibition of cell proliferation was calculated by the following formula:

$$\begin{array}{ll} \mathrm{Percentage}\;\mathrm{inhibition}= & \left(\mathrm{Absorbanc}e_{\mathrm{control}}-\mathrm{Absorbanc}e_{\mathrm{treated}}\right)\\ & \;/\mathrm{Absorbanc}e_{\mathrm{control}}\times 100 \end{array}$$

### Estimation of antioxidant enzymes

#### Preparation of cell lysate

MCF-7 cells were seeded in a 6-well plate (1.5 × 10^6^ cells/well) in RPMI-1640 supplemented with 5% FBS. After 24 h, cells were treated with the ethyl acetate extract of *C. sativum* roots at a final extract concentration of 200 μg/ml (IC_50_ concentration as determined from the MTT assay) in the well. DMSO was used to replace extracts in the untreated control samples. Cells were treated for 0, 6, 9, 15, 24 and 48 h. Following incubation, cells were washed with PBS, harvested using a cell scraper and collected for centrifugation at 1,000 rpm for 5 min at 4°C. The supernatant was discarded and the cell pellet resuspended in cold PBS. The number of cells was determined by the trypan blue dye exclusion method. Cells were then lysed while on ice using a sonicator and centrifuged for 10 min at 6,000 × *g* at 4°C. The supernatant was used in antioxidant enzyme assays.

#### Superoxide dismutase (SOD) assay

The SOD activity in MCF-7 cells treated with test samples was assayed in triplicate using the superoxide dismutase assay kit by Cayman Chemical (USA). The assay uses a tetrazolium salt for detection of superoxide anion radicals generated by xanthine oxidase. The assay was performed according to the manufacturer’s protocol. SOD activity was calculated using the following equation:

$$\begin{array}{ll} \;\mathrm{SOD}\left(U/\mathrm{ml}\right)= & \left[\left(\mathrm{sampleLR}-y\mathrm{int}\mathrm{ercept}\right)/\mathrm{slope}\right]\\ & \times \left(0.23\mathrm{ml}/0.01\mathrm{ml}\right) \end{array}$$

One unit is defined as the amount of enzyme needed to exhibit 50% dismutation of the superoxide anion radical. SOD activity was expressed in U/ml per 10^6^ cells.

#### Glutathione peroxidase (GPx) assay

The GPx activity in MCF-7 cells treated with test samples was assayed in triplicate using the glutathione peroxidase assay kit by Cayman Chemical (USA). This assay measures GPx activity indirectly by a coupled reaction with glutathione reductase. The assay was performed according to the manufacturer’s protocol. GPx activity was calculated using the following equation:

$$\begin{array}{l} \mathrm{GPx}\;\mathrm{activity}\left(\mathrm{nmol}/\min/\mathrm{ml}\right)\\ \;=\left(\mathrm{change}\;\mathrm{in}\;\mathrm{absorbance}\;\mathrm{per}\min/0.00373\mu M^{-1}\right)\\ \;\times \left(0.19\mathrm{ml}/0.02\mathrm{ml}\right) \end{array}$$

One unit is defined as the amount of enzyme that causes the oxidation of 1 nmol of NADPH to NADP^+^ per min at 25°C. GPx activity was expressed in nmol/min/ml per 10^6^ cells.

#### Catalase (CAT) assay

The CAT activity in cells treated with test samples was assayed in triplicate using the catalase assay kit by Cayman Chemical (USA). The assay is based on the reaction of CAT with methanol in the presence of H_2_O_2_ which produces formaldehyde. Formaldehyde is measured colorimetrically using 4-amino-3-hydrazino-5-mercapto-1,2,4-triazole (Purpald) as the chromogen. The assay was performed according to the manufacturer’s protocol. CAT activity was calculated using the following equation:

$$\begin{array}{l} \mathrm{Catalase}\;\mathrm{activity}\left(\mathrm{nmol}/\min/\mathrm{ml}\right)=\\ \;\mathrm{formaldehyde}\;\mathrm{concentration}\;\mathrm{of}\;\mathrm{sample}\left(\mathrm{\mu M}\right)/20\min \end{array}$$

One unit is defined as the amount of enzyme that will cause the formation of 1 nmol of formaldehyde per min at 25°C. The CAT activity was expressed in nmol/min/ml per 10^6^ cells.

### Colorimetric assays of caspase-3, -8 and -9

A time- and dose-dependent study of caspase-3, -8, and -9 activities in MCF-7 cells was performed in triplicate using Caspase-3/CPP32, FLICE/Caspase-8 and Caspase-9 colorimetric assay kits by BioVision (USA). The test assays for the activities of caspase-3, -8 and -9 that recognise the amino acid sequence, DEVD, IETD, and LEHD, respectively. The assay is based on spectrophotometric detection of the chromophore *p*-nitroanilide (*p*NA), which is released from labeled substrates after cleavage by caspase. In a 6-well plate, MCF-7 cells (1.5 × 10^6^ cells/well) were seeded with RPMI-1640 containing 5% FBS. After 24 h of incubation, cells were treated with *C. sativum* (root) ethyl acetate extract at a final concentration of 200 μg/ml (IC_50_) and 276 μg/ml (IC_70_) in the well. DMSO was used in place of the extract for control wells. Colchicine for caspase-3 and mitomycin C for caspases-8 and -9 at 1 μM were used as positive controls. Cells were treated for 6 and 24 h and then harvested. Caspase activities of cell lysates were assayed according to the manufacturer’s protocol. Briefly, in a 96-well plate, 50 μg of protein sample was diluted in 50 μl of cell lysis buffer and 50 μl of 2× reaction buffer (containing 10 mM DTT) was added into each well. For the caspase-3 assay, 5 μl of 4 mM DEVD-*p*NA substrate (200 μM final concentration) was added into wells and the mixture incubated at 37°C for 2 h. For analysis of caspase-8 and caspase-9, the substrates IETD-*p*NA and LEHD-*p*NA, respectively, were used. The absorbance of the wells was read at 405 nm. The data was presented as fold change.

### Cell cycle analysis

To determine the distribution of extract-treated MCF-7 cells in different phases of the cell cycle, DNA content in cells was detected by propidium iodide (PI) staining and flow cytometry. MCF-7 cells were cultured in 75 cm^2^ flasks at a density of 4 × 10^6^ cells in 5% FBS. After 24 h of incubation, cells were treated with the ethyl acetate extract of the roots, at a final concentration of 276 μg/ml (IC_70_) in the flask for another 24, 48 and 72 h. Untreated control samples were performed using DMSO to replace extracts. The cells were collected, washed and fixed in 70% ethanol at -20°C overnight. Cells were then washed in PBS, stained in 500 μl of PI/RNase staining buffer (Becton Dickinson, USA) and incubated in RT for 15 min in the dark. Cell cycle phase distribution was determined using BD FACSCanto II flow cytometer instrument and BD FACSDiva software (Becton Dickinson, USA). A total of 15,000 events per sample were collected for analysis. The fluorescence intensity of the sub-G_1_ cell fraction represents the apoptotic cell population.

### Scratch motility assay

MCF-7 cells (3.5 × 10^5^ cells/well) were seeded in a 24-well plate and grown for 24 h. The confluent cell monolayer was then scratched vertically with a pipette tip, washed twice with PBS and incubated with media containing *C. sativum* (root) ethyl acetate extract (0, 100, 150, 200, 250 and 300 μg/ml) with 5% FBS. H_2_O_2_ was added into each well at a final concentration of 1 μM in the cell suspension to stimulate the proliferation and migration of MCF-7 cells. The number of cells in the denuded area were photographed and counted at 0- and 24-h incubation. The experiment was performed in triplicate. The percent inhibition was calculated as described by Sato & Rifkin [20]. Percent inhibition = 100 – [(cell no. in denuded area of sample / cell no. in denuded area of control) × 100].

### Comet assay

In a 12-well culture plate, 3 T3-L1 mouse fibroblasts (1 × 10^5^ cells/well) were cultured in DMEM with 10% FBS for 24 h. The cells were pre-treated with the root ethyl acetate extract at concentrations of 100–400 μg/ml in the well for another 24 h. The control was performed using DMSO to replace extracts. After pre-treatment, cells were exposed to 100 μM of H_2_O_2_ (final concentration in the cell suspension) for 60 min on ice to induce DNA damage. Following H_2_O_2_ exposure, cells were harvested using a cell scraper, centrifuged and resuspended in 1 ml of PBS for use in comet assay [21]. Briefly, 25 μl of the cell suspension was mixed with 75 μl of 0.6% low melting agarose. The suspension was spread on a frosted microscopic slide pre-coated with 250 μL of 0.8% normal melting agarose, covered with a cover slip, and then allowed to solidify on ice for 10 min. The cover slips were removed and the slides were immersed in cold lysis solution containing 1% sodium dodecyl sulfate, 2.5 M NaCl, 100 mM Na_2_EDTA, 1% Triton X-100 and 10% DMSO (with the DMSO added just before use) for one hour at 4 °C in the dark. Then, slides were arranged in an electrophoresis tank filled with pre-chilled electrophoretic buffer (1 mM Na_2_EDTA and 300 mM NaOH) and incubated for 20 min. Electrophoresis was conducted in the same buffer, in a horizontal chamber, at 25 V (300 mA) for 20 min using a power supply (CBS Scientific company, USA). The slides were washed with 0.4 M Tris–HCl (pH 7.5) and stained with 20 μg/ml ethidium bromide for viewing under a BX50 fluorescence microscope (Olympus, Japan). Electrophoresis of the samples separates intact DNA from damaged fragments. The comet tail length is associated with DNA damage. Greater tail length signifies greater DNA damage [21]. A total of 50 individual cells were screened per slide [22]. The assay was carried out in triplicate. The comet tail length was measured using an ocular micrometer.

Results were expressed in% DNA protection calculated by the following formula:

$$\begin{array}{ll} \;\mathrm{DNA}\;\mathrm{protection}\left(\%\right)= & \left(\mathrm{tail}\;\mathrm{lengt}h_{\mathrm{control}}-\mathrm{tail}\;\mathrm{lengt}h_{\mathrm{treatment}}\right)\\ & \;/\mathrm{tail}\;\mathrm{lengt}h_{\mathrm{control}}\times 100 \end{array}$$

### Identification of compounds

#### High performance liquid chromatography (HPLC) analysis

The *C. sativum* (root) ethyl acetate extract was subjected to acid hydrolysis to release free polyphenols from their glycosides according to the method of Nuutila, Kammiovirta, & Oksman-Caldentey [23], with slight modifications. Briefly, 20 mg of dried extract in 0.4 ml of 6 N HCl and 1.6 ml of HPLC grade methanol with 20 mM butylated hydroxytoluene (BHT) as antioxidant was heated at 90°C for 2 h. The mixture was centrifuged at 10,000 rpm for 5 min and the supernatant was filtered through a 0.2 μm syringe filter and stored at 4°C for HPLC analysis. HPLC analysis was performed using a SPD-20A HPLC system (Shimadzu, Japan). Reverse phase separation was performed at 40°C using a Purospher STAR RP-18 endcapped column (5 μm) (Merck, Germany). The mobile phase consisted of trifluoroacetic acid in ultrapure water at pH 2.6 (solvent A) and acetonitrile (solvent B). The gradient program consisted of: 0% to 12.5% B for 2.5 min, 12.5% to 100% B for 17.5 min and 100% B for 10 min. The flow rate was kept at 1 ml/min and injection volume was 10 μl. The chromatogram peaks were detected at 254 nm. Data acquisition and processing was performed using LCsolution software (Shimadzu, Japan). The compounds were identified by comparing the retention times of peaks with standards. The extract was then spiked with the standards to confirm their presence. Unidentified peaks were collected manually and the mobile phase was air dried. The dried fraction (referred to as fraction S1) was stored at 4°C for analysis with GC-MS.

#### Gas chromatography–mass spectrometry (GC-MS) analysis

Prior to GC-MS analysis, chemical derivatisation was performed to reduce the polarity of functional groups by reconstituting 400 μg of the dried fraction S1 (unidentified peaks collected from HPLC) with 500 μl of HPLC grade ethyl acetate and 20 μl of N,O-bis(trimethylsilyl)trifluoroacetamide (BSTFA), and heated at 70°C for 40 min. The GC-MS analyses were carried out in a GCMS-QP2010 system (Shimadzu, Japan) fitted with a ZB-5 (30 m × 0.25 mm i.d.) capillary column (Phenomenex, USA). The carrier gas was helium with a flow rate of 1.08 ml/min. The column temperature was set at 100°C for 5 min, 100–275°C at 10°C/min, and finally held for 20 min in 275°C. Sample volume injected was 1 μl with a split ratio of 2:1. The injector temperature was 250°C and the detector temperature was 290°C. The MS operating parameters were: ionisation potential, 70 eV, ion source temperature, 200°C, solvent delay, 3.0 min, scan speed, 2500 amu/s, scan range, 40–500 amu and detector voltage, 1.5 kV. Compound identification was verified based on mass spectral data by computer matching with Wiley 229, NIST 107, NIST 21 and PMW_tox2 libraries.

### Statistical analysis

Data are presented as mean ± standard deviation (SD). Statistical analyses were performed by one-way analysis of variance (ANOVA) with Tukey’s multiple comparisons and the Student’s *t*-test. A P-value of *<* 0.05 was considered statistically significant. Pearson correlation coefficient was used to assess the correlation between TPC, FRAP and DPPH radical scavenging activity. SPSS, version 18.0 (Chicago, Ill, USA) and Microsoft Excel 2007 (Roselle, Ill, USA) statistical software were used for the statistical and graphical evaluations.

## Results

### Total phenolic content

The amount of total phenolics in extracts of *C. sativum* ranged from 1.73 ± 0.49 to 31.38 ± 2.75 mg GAE/g (Table 1). In both of the plant parts (root and leaves), the ethyl acetate extracts showed the highest TPC values of 31.38 ± 2.75 mg GAE/g (root) and 24.57 ± 0.70 mg GAE/g (leaf and stem).

**Table 1:** **Phenolic content, ferric reducing antioxidant power and DPPH radical scavenging activity of *C. sativum *** **extracts**

**Plant extract/positive controls**	**Total phenolic content (mg GAE/g)**	**FRAP value (mmol/g)**	**DPPH radical scavenging activity (μg/ml)**
*C. sativum* (root) Ethyl acetate	31.38 ± 2.75	0.129 ± 0.007	2348.3 ± 184.1
*C. sativum* (leaf and stem)	24.57 ± 0.70 (Ethyl acetate)	0.136 ± 0.008 (Dichloromethane)	1335.0 ± 37.7 (Aqueous)
*Positive control*			
Rutin	649.93 ± 13.34	1.789 ± 0.214	42.7 ± 2.3
Quercetin	1275.62 ± 56.03	14.444 ± 0.934	22.2 ± 0.9

Each value is expressed as mean ± SD (n = 3).

IC_50_ values are presented for the DPPH radical scavenging activity.

All values are significantly different at P < 0.05 compared to the negative control as tested by the Student’s *t*-test.

### Ferric reducing antioxidant power

Among *C. sativum* root extracts, the ethyl acetate extract had the highest FRAP value of 0.129 ± 0.007 mmol/g (Table 1). As for the leaf and stem, highest FRAP value was seen in the dichloromethane extract (0.136 ± 0.008 mmol/g).

### DPPH radical scavenging activity

The leaf and stem aqueous extract had the lowest IC_50_ value of 1335.0 ± 37.7 μg/ml (Table 1). As for the root, the ethyl acetate extract which had highest FRAP value, also displayed highest DPPH radical scavenging activity (IC_50_ = 2348.3 ± 184.1 μg/ml) among root extracts.

### Correlation analyses of TPC with FRAP and DPPH radical scavenging activity

The relationship between phenolic content with FRAP and DPPH scavenging activity of plant extracts were evaluated by Pearson correlation analyses. A strong and statistically significant positive correlation was identified between the phenolic content and FRAP values of the root (*r* = 0.982, P < 0.01) while a weak positive correlation was seen between its TPC and DPPH scavenging activity (*r* = 0.663, P < 0.01) (Table 2). As for the leaf and stem, significant positive correlation was observed between its TPC and DPPH scavenging activity (*r* = 0.906, P < 0.01).

**Table 2:** **Correlation analyses of the total phenolic content and antioxidant activities of *C. sativum *** **extracts**

**Plant**	**Pearson correlation *(r) *** **value**	
	**TPC/FRAP**	**TPC/DPPH**
*C. sativum* (root)	0.982*	0.663*
*C. sativum* (leaf and stem)	0.300	0.906*

TPC, total phenolic content; FRAP, ferric reducing antioxidant power; DPPH, 1,1-diphenyl-2-picryl hydrazyl radical scavenging activity.

* Correlation is significant at the 0.01 level.

### Anti-proliferative activity

The anti-proliferative activity of *C. sativum* extracts was investigated on the breast adenocarcinoma cell line, MCF-7, using the MTT assay. Results were expressed as 20% inhibitory concentration (IC_20_) and 50% inhibitory concentration (IC_50_) (Table 3). Among the five extracts analyzed from *C. sativum* root, the ethyl acetate extract exhibited the best antiproliferative activity with the lowest IC_50_ value of 200.0 ± 2.6 μg/ml. Among extracts of the leaf and stem, the hexane extract showed the lowest IC_50_ value (432.3 ± 41.0 μg/ml). As the root ethyl acetate extract displayed the best anti-proliferative activity, subsequent analyses focused on this extract and its effect on MCF-7 cells. The ethyl acetate extract of the root showed less toxicity on the nonmalignant human breast epithelial cell line, 184B5, with an IC_50_ value of 317.0 ± 9.6 μg/ml compared to MCF-7 cells (Table 3).

**Table 3:** **Summary of the anti-proliferative activities of *C. sativum *** **extracts on MCF-7 and 184B5 cell lines**

**Plant extract**	**MCF-7**		**184B5**	
	**IC**_**20 **_**(μg/ml)**	**IC**_**50 **_**(μg/ml)**	**IC**_**20 **_**(μg/ml)**	**IC**_**50 **_**(μg/ml)**
*C. sativum* (root) Ethyl acetate	100.0 ± 1.7	200.0 ± 2.6	174.3 ± 26.0	317.0 ± 9.6
*C. sativum* (leaf and stem) Hexane	138.0 ± 8.7	432.3 ± 41.0	ND	ND

Each value is expressed as mean ± SD (n = 3).

ND = Not Detected.

### Estimation of antioxidant enzymes

**Figure 1:**
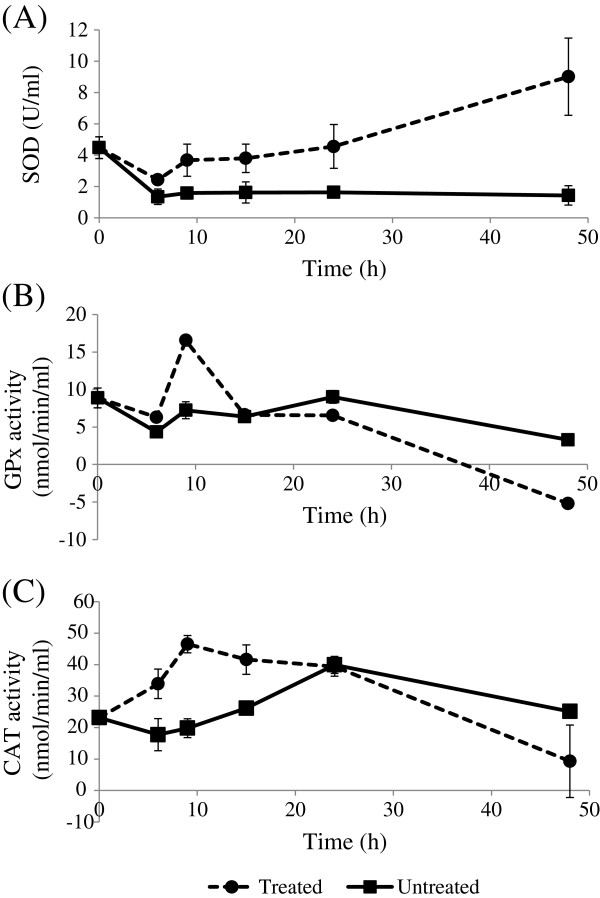
**Antioxidant enzyme activities in untreated and treated MCF-7 cells with *C. sativum *root extract.** MCF-7 cells (1.5 × 10^6^) were treated with *C. sativum* root ethyl acetate extract at 200 μg/ml (IC_50_ concentration) for 0, 6, 9, 15, 24 and 48 h. DMSO was used to replace the extract in untreated cells. (**A**) Superoxide dismutase (SOD) activity is expressed in U/ml per 10^6^ cells, while (**B**) glutathione peroxidase (GPx) and (**C**) catalase (CAT) activities are expressed in nmol/min/ml per 10^6^ cells. Results are expressed as mean ± SD (n = 3).

Antioxidant enzyme activities in root ethyl acetate extract*-*treated and -untreated MCF-7 cells were estimated. Treated cells showed increasing SOD activity (from 6–48 h) compared to the untreated cells (Figure 1A). The GPx activity in treated cells increased from 6–9 h and then decreased from 24–48 h (Figure 1B) while CAT activity increased from 0–9 h and then decreased until 48 h (Figure 1C).

### Activity of caspase-3, -8 and -9

The results indicated that activities of caspases-3, -8 and -9 were significantly enhanced in MCF-7 cells treated with the root extract for 24 h compared to untreated cells (Table 4). The activities of caspase-3, -8, and -9 in cells treated with 200 μg/ml of extract for 24 h increased by 1.20, 1.16 and 1.12 fold, respectively, compared to untreated cells. At treatment with 276 μg/ml of extract for 24 h, the activities of caspase-3, -8, and -9 increased by 1.28, 1.21 and 1.30 fold, respectively, compared to untreated cells.

**Table 4:** **Activities of caspase-3, -8 and -9 in MCF-7 cells treated with *C. sativum *** **root extract**

**Treatment**	**Fold change compared to control**		
	**Caspase-3**	**Caspase-8**	**Caspase-9**
Control	1.00 ± 0.00	1.00 ± 0.00	1.00 ± 0.00
***6 h***			
200 μg/ml	0.87 ± 0.02	0.83 ± 0.00	0.84 ± 0.03
276 μg/ml	0.86 ± 0.03	0.87 ± 0.03	0.88 ± 0.04
Positive control	0.91 ± 0.01	0.87 ± 0.01	0.87 ± 0.02
***24 h***			
200 μg/ml	1.20 ± 0.02*	1.16 ± 0.02*	1.12 ± 0.03*
276 μg/ml	1.28 ± 0.02*	1.21 ± 0.03*	1.30 ± 0.01*
Positive control	1.06 ± 0.01*	1.06 ± 0.02*	1.07 ± 0.02*

The positive controls used were colchicine (caspase-3) and mitomycin C (caspase-8 and -9) at 1 μM.

Values are expressed as fold change compared to control, mean ± SD (n = 3).

*Values are significantly different at P < 0.05 compared to control as tested by the Student’s *t*-test.

### Cell cycle analysis

**Figure 2:**
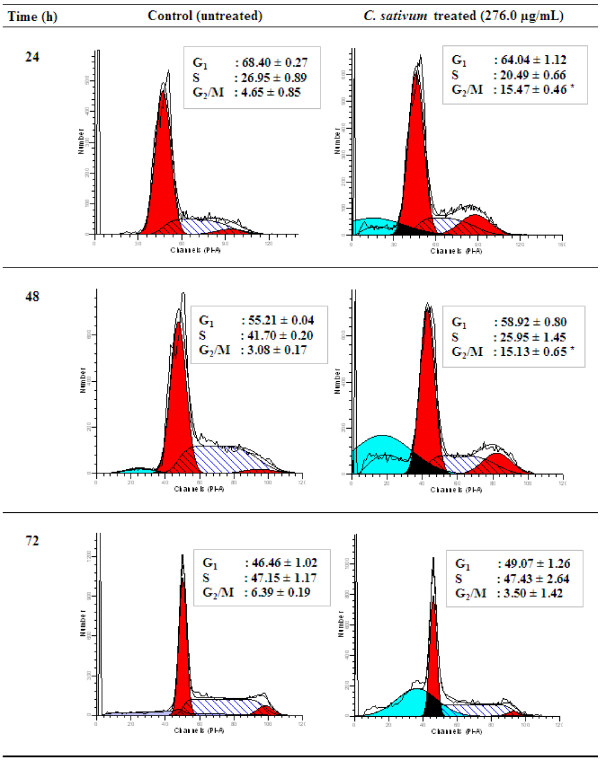
***C. Sativum *root extract induces cell cycle arrest at G**_**2**_**/M phase in MCF-7 cells.** The cell cycle distribution was determined by propidium iodide staining and flow cytometry. MCF-7 cells were treated with the root ethyl acetate extract (276.0 μg/ml) for 24, 48 and 72 h. Untreated cells at each time point were included as controls. The DNA histogram shows the distribution and the percentage of cells in phases of the cell cycle. Results are the mean ± SD of 3 independent experiments. *P < 0.01 compared to the control as tested by the Student’s *t*-test. Each DNA histogram represents one of the three independent experiments.

Flow cytometric analysis of DNA content and cell cycle distribution was performed to determine the ability of *C. sativum* root extract to induce MCF-7 cell cycle arrest and apoptosis. The sub-G1 population of cells (apoptotic population) increased significantly (P < 0.01) in a time-dependent manner as compared to the control (Table 5). The decrease in the S phase population was accompanied by significantly increased G_2_/M phase population (P < 0.01) after 24 and 48 h treatment compared to the control (Figure 2), indicating cell cycle arrest at the G_2_/M phase in treated cells. At 72 h, treated cells had no increment in the G_2_/M population (Figure 2) but increased in the sub-G1 population compared to the control (Table 5), suggesting that cells were arrested at the G_2_/M phase followed by significant apoptotic cell death over time.

**Table 5:** **Sub-G**_**1 **_**populations in untreated (control) and treated MCF-7 cells with *C. sativum *** **root extract**

**Time (h)**	**Sub-G**_**1 **_**(%)**	
	**Control**	**Treated**
24	0.73 ± 0.60	18.53 ± 1.25 ^*^
48	2.59 ± 2.16	35.67 ± 2.61 ^*^
72	0.78 ± 1.34	42.46 ± 2.06 ^*^

Each value is expressed as mean ± SD (n = 3).

*P < 0.01 compared to the control as tested by the Student’s *t*-test.

### Inhibition of H_2_O_2_-induced MCF-7 cell migration using the scratch motility assay

**Figure 3:**
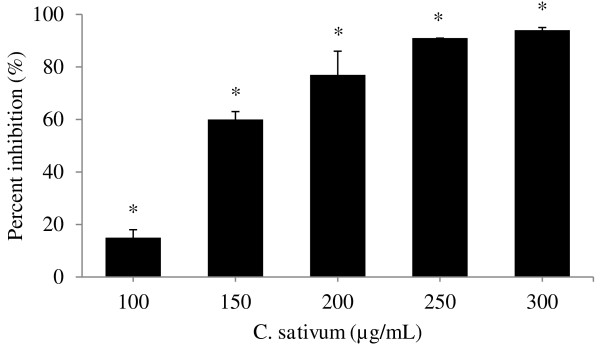
**Inhibition of H**_**2**_**O**_**2**_**-induced MCF-7 cell migration by *C. sativum *root ethyl acetate extract.** Results are presented as mean ± SD (n = 3). * P < 0.05 compared to the control (without extract) as tested by the Student’s *t*-test.

The scratch motility assay displayed the ability of the root extract to suppress H_2_O_2_-induced migration of MCF-7 cells in a denuded area. The extract inhibited cell migration induced by H_2_O_2_ following a dose-dependent pattern (Figure 3). At 150 μg/ml of extract, inhibition of MCF-7 migration in the denuded area was 60 ± 3%. At higher extract concentrations, the percent inhibitions of MCF-7 migration increased up to 91 ± 0% (250 μg/ml) and 94 ± 1% (300 μg/ml).

### DNA protective activity

The protective effect of the *C. sativum* root extract on 3 T3-L1 cells from H_2_O_2_-induced DNA damage was investigated using the comet assay. Fibroblasts pre-treated with the extract at 100–400 μg/ml showed a significant dose-dependent increase in DNA protection (P < 0.05) compared to the control (without extract treatment) (Table 6). At 400 μg/ml of extract pretreatment, DNA protection was 21.5 ± 6.6%.

**Table 6:** **Protection from H**_**2**_**O**_**2**_**-induced DNA damage in 3 T3-L1 fibroblasts pre-treated with *C. sativum *** **root extract**

***C. sativum *** **(μg/mL)**	**DNA protection (%)**
100	3.4 ± 4.7*
200	6.5 ± 5.5*
300	13.4 ± 6.1*
400	21.5 ± 6.6*

Means ± SD are presented (n = 3).

* P < 0.05 compared to control (without extract treatment), as tested by the Student’s *t*-test.

### Identification of compounds in *C. sativum* root ethyl acetate extract

**Figure 4:**
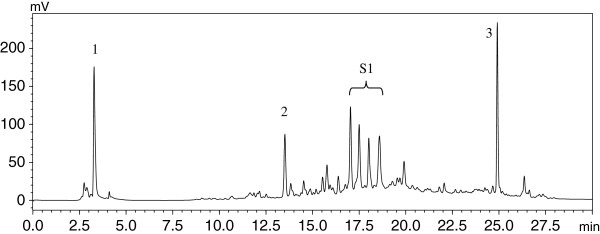
**HPLC chromatogram of *C. sativum *root extract.** Reverse phase separation was performed at 40°C using a Merck Purospher STAR RP-18 endcapped column (5 μm). The mobile phase consisted of trifluoroacetic acid in water at pH 2.6 (solvent A) and acetonitrile (solvent B). The gradient program consisted of: 0% to 12.5% B for 2.5 min, 12.5% to 100% B for 17.5 min and 100% B for 10 min. The flow rate was kept at 1 ml/min and injection volume was 10 μl. The eluted peaks were monitored at 254 nm. 1: ascorbic acid; 2: *p*-coumaric acid; 3: butylated hydroxytoluene; S1: unidentified peaks collected for GC-MS analysis. (BSTFA = N,O-bis(trimethylsilyl)trifluoroacetamide).

The compounds in *C. sativum* root ethyl acetate extract were identified by HPLC and GC-MS analyses. Figure 4 shows the HPLC chromatogram of *C. sativum* (root) ethyl acetate extract. Ascorbic acid and *p*-coumaric acid were detected in the extract. Peak 3 is butylated hydroxytoluene, an antioxidant added to *C. sativum* (root) ethyl acetate sample during extract preparation for HPLC analysis. Several peaks that did not correspond to the standards used in the HPLC analysis were observed in the chromatogram between retention times 15–20 min. The unidentified peaks marked as fraction S1 (Figure 4) were collected and analysed by GC-MS. Cinnamic acid, 4,4,5,7,8-pentamethyl-3,4-2H-isocoumarin-3-one, 1,3,4 tris(trimethylsilyloxy)octadecan-2-amine and L-valine were identified from GC-MS analyses. These four compounds most possibly represent the four unidentified peaks of fraction S1 (Figure 4).

## Discussion

*C. sativum* has been popularly used as a dietary herb to flavor food and also treat ailments and to promote health and well-being. In this study, the antioxidant, anticancer and MCF-7 cell migration inhibitory activities of *C. sativum* were investigated. The DNA protective ability in normal cells was also investigated.

Antioxidant activities of *C. sativum* reported in literature mostly focused on the aerial parts of the herb. In this study, we showed the antioxidant activities of *C. sativum* root, and the leaf and stem. As antioxidant activities can be affected by location and growth conditions of the plant, antioxidant data on the leaf and stem is appropriate. The nature of the extracting solvent is one of the most important factors in the extraction of antioxidants and bioactive compounds. Hence, we used a range of non-polar to polar solvents to sequentially extract compounds from *C. sativum* and the extracts were tested for antioxidant potential and inhibitory activities on cancer cells. Plant antioxidant studies reported that ethyl acetate allowed for the selective removal of non-phenolic compounds and achieved the highest phenolic content [24]. The ethyl acetate extracts of *C. sativum* leaf and seed have been reported with highest phenolic content [14,15]. Likewise, our study showed highest phenolic content in ethyl acetate extracts of *C. sativum* parts, with the highest TPC in the root. The root ethyl acetate extract also had highest FRAP and DPPH scavenging activities among extracts of the root. Antioxidant activities of medicinal plants have been attributed to their phenolic content [25]. Our correlation analyses showed positive correlation between the phenolic content of *C. sativum* (root) with FRAP and DPPH scavenging activities. This implies that phenolic compounds in *C. sativum* root extracts have reductive abilities and may provide antioxidative protection in actual biological systems by donating electrons to radicals. This study also showed that the leaf and stem dichloromethane and aqueous extract had highest FRAP and DPPH scavenging activity, respectively. In a different study, the aqueous extract of *C. sativum* leaf and shoots exhibited antioxidant activity in a *β*-carotene/linoleic acid model [26]. Our study together with those reported in literature show that the various parts of *C. sativum* have antioxidant properties that protect cells from the adverse effects of oxidative stress caused by ROS.

Each extract of *C. sativum* showed different anti-proliferative effects on the MCF-7 cell line, which may be due to extract phytodiversity, different mechanisms of action by compounds in extracts, and the various susceptibility levels of cell lines to extracts [27]. In this study, the root ethyl acetate extract which displayed the highest phenolic content, also showed the best anti-proliferative activitiy in MCF-7 cells. Hence, we selected the root ethyl acetate extract to further analyze its anticancer effects on antioxidant enzymes, caspase activity, cell cycle arrest, and inhibition of cell migration in MCF-7 cells. The protective effect of the extract on H_2_O_2_-induced DNA damage was determined using non-cancerous 3 T3-L1 fibroblasts. Although *C. sativum* (leaf and stem) dichloromethane and aqueous extracts had higher FRAP and DPPH scavenging activity, respectively, compared to the root extract, these extracts exhibited weak antiproliferative activity and were thus not selected for subsequent assays.

High levels of H_2_O_2_ can produce cancer cell death [6]. We showed that MCF-7 cells treated with *C. sativum* root ethyl acetate extract displayed increasing SOD activity over 48 h of treatment while GPx and CAT activities decreased from 24–48 h. Antioxidant enzymes are involved in the direct elimination of ROS in cells. SOD converts superoxide anion to H_2_O_2_, while GPx and CAT convert H_2_O_2_ to water and oxygen. As the H_2_O_2_-detoxifying enzymes, GPx and CAT activities decreased with extract treatment, high levels of H_2_O_2_ produced by the increasing SOD activity possibly led to H_2_O_2_ accumulation, leading to MCF-7 cancer cell death [6]. A possible explanation for the decrease in GPx and CAT activity in treated cells from 24–48 h is due to increasing ROS. CAT can be downregulated by ROS [28] while GPx can be inactivated by peroxides and hydroxyl radicals [29]. Rashad, El-Sayed, Mohamed, & Ali [30] reported that quinoline derivatives inhibited the growth of MCF-7 cells by similarly increasing the activity of SOD and decreasing CAT and GPx activities, accompanied by a high production of H_2_O_2_ and other free radicals which caused cancer cell death.

There is an added feature in the root extract causing MCF-7 cell death by H_2_O_2_ accumulation. Experimental evidence has shown that cancer cells are more susceptible to H_2_O_2_-induced cell death compared to normal cells [31]. There is a threshold of H_2_O_2_ above which cells cannot survive. Cancer cells have higher levels of H_2_O_2_ than normal cells. A slight elevation of H_2_O_2_ in cancer cells causes their H_2_O_2_ levels to increase above the toxic threshold, making these cells more susceptible to H_2_O_2_-induced cell death [6]. This is shown in our study where the root extract had lower toxicity on the nonmalignant human breast epithelial cell line, 184B5 compared to MCF-7 breast cancer cells. A successful anticancer agent causes cancer cell death without damaging normal cells excessively. A review by Burdock & Carabin [32] reported on the safety of coriander seed essential oil as an added food ingredient.

The caspase cascade signaling system is an important component in the process of apoptosis. The activation of the downstream pathways of caspase-8 varies with different cell types. In Type I cells (cells of some lymphoid cell lines), the activation of caspase-8 directly activates procaspase-3. In Type II cells (cells other than Type I cells), the activation of caspase-8 causes caspase-9 activation, which then induces cleavage of procaspase-3 [33]. As caspase-9 (mitochondrial pathway) is known to be activated following caspase-8 (death receptor pathway) activation in MCF-7 cells (Type II cells) [34], it is postulated that caspase-8 activation is an initiating event in the apoptotic cell death induced by the root extract which led to the higher activities of caspase-9 and -3. The activation of caspase-3 is an important downstream step in the apoptotic pathway. Caspases-3, -8 and -9 are the main executors of apoptosis [35]. Our study suggest that *C. sativum* root extract inhibited MCF-7 breast cancer cells by the death receptor and mitochondrial apoptotic pathways as demonstrated by significantly increased caspases-3, -8 and -9 activities compared to the control. Jänicke [36] reported that the MCF-7 cell line from ATCC lacks functional caspase-3. However, there are studies that reported the presence of caspase-3 in MCF-7 [37,38]. In our study, activation of caspase-3 was observed. The different reports on the presence of caspase-3 in MCF-7 could be due to the different variants of MCF-7 cells used [39].

From the flow cytometric analysis, we further confirm that *C. sativum* root extract induced apoptotic cell death in MCF-7 cells, characterized by an increase in sub-G_1_ cells [40]. The extract also caused cell cycle arrest at the G_2_/M phase. As avoidance of cell cycle arrest is a common alteration in cancer, agents that are able to induce cell cycle arrest can act as a barrier to cancer and are increasingly used in combination with conventional cytotoxic drugs to improve anticancer efficacy and overcome drug resistance [41], thus displaying the potential use of *C. sativum* root to enhance conventional chemotherapy.

From the scratch motility assay, we showed that *C. sativum* root extract is able to inhibit H_2_O_2_-induced MCF-7 cancer cell migration, indicating its potential in preventing metastasis. H_2_O_2_ was used in this experiment to induce migration of MCF-7 cells in the denuded area. The concentration of H_2_O_2_ (1 μM) used in this assay has been previously tested in our laboratory and showed increased cell migration and proliferation [22]. Antioxidants in the root extract can decrease H_2_O_2_ levels and may be associated with the prevention of cancer cell proliferation and migration [6].

The comet assay is a quick, simple and sensitive method for the evaluation of DNA damage, mainly single-strand and double-strand breaks in individual cells. H_2_O_2_ produces reactive hydroxyl radicals which can induce strand breaks associated with DNA damage. We showed that *C. sativum* root extract protected 3 T3-L1 cells against H_2_O_2_-induced DNA damage, suggesting protection from free radical induced carcinogenesis, i.e., chemoprotective activity. A study on spices (ginger, caraway, cumin, cardamom, star anise and fennel) showed a strong positive correlation between DNA protection and phenols [22]. This implies that phenolics from the root extract may have contributed to the observed DNA protective activity. Phenolics might lower H_2_O_2_ levels or hydroxyl radicals by increasing the levels of H_2_O_2_-detoxifying enzymes in cells [15]. From other reports, *C. sativum* had marked anti-genotoxic and anti-carcinogenic activities against several genotoxicants, like 4-nitro-*o*-phenylenediamine, *m*-phenylenediamine and 2-aminofluorene [42], and protected against carbon tetrachloride-induced hepatotoxicity in rats [8].

We identified ascorbic acid, the phenolics *p*-coumaric acid and cinnamic acid, 4,4,5,7,8-pentamethyl-3,4-2H-isocoumarin-3-one, 1,3,4-tris(trimethylsilyloxy)octadecan-2-amine and the amino acid L-valine in the *C. sativum* root ethyl acetate extract. Acid hydrolysis was performed on the extract before HPLC analysis. Although hydrolysis may raise questions with respect to sample preparation, analyte stability and recoverability, this step cleaves ester bonds and simplifies the analysis by reducing the number of derivatives [43]. Fraction S1 was derivatized with BSTFA to increase its volatility for GC-MS analysis. Silylation has become the major derivatization technique as the reaction is simple and the byproducts of these reactions are extremely volatile, elute very early, and do not interfere with the analysis [44].

Previous studies reported the presence of ascorbic acid [45], *p*-coumaric acid [46], cinnamic acid [47] and valine [11] in *C. sativum*. Isocoumarins such as coriandrones A - E, coriandrin and dihydrocoriandrin have been reported in *C. sativum*[46]. In this study, we identified 4,4,5,7,8-pentamethyl-3,4-2H-isocoumarin-3-one in *C. sativum* root, which was also reported in the essential oil of *Dictamnus dasycarpus* root bark [48]. HPLC fingerprinting of ethanolic extract of *C. sativum* leaves showed the presence of iso-quercetin and quercetin [49]. In a recent study, the essential oil of *C. sativum* analyzed by GC-MS revealed 39 and 38 components identified from the leaves and stems, respectively. Among the major components reported in the leaves were cyclododecanol (23.11%), tetradecanal (17.86%), 2-dodecenal (9.93%) and 1-decanol (7.24%). The major components in the stems were phytol (61.86%), 15-methyltricyclo[6.5.2(13,14),0(7,15)]-pentadeca-1,3,5,7,9,11,13-heptene (7.01%), dodecanal (3.18%), and 1-dodecanol (2.47%) [50].

Extensive study has been done on ascorbic acid as an antioxidant and pro-oxidant with anticarcinogenic and anticancer properties [51]. Ascorbic acid has been shown to be cytotoxic to MCF-7 and HT-29 cells [52,53] and to induce cell death through the generation of H_2_O_2_[54]. Generation of oxidative stress due to H_2_O_2_ is associated with the arrest of cancer cell proliferation and triggering of apoptosis [55]. Ascorbic acid has been reported to induce cell cycle arrest at the G_2_/M phase [56] and mitigate tumor metastasis [57]. We observed similarities in the anticancer effects of ascorbic acid reported in literature with those exhibited by the ethyl acetate extract of *C. sativum* root in this study. Supported by literature and from the results of our study, we postulate that ascorbic acid contributed to the anticancer and antioxidant activities of *C. sativum* root observed in this study. However, the role of the other compounds reported in the root should not be disregarded as the mixture of compounds in an extract and their possible additive or synergistic action could be responsible for the bioactivities produced by an extract.

Though the anti-proliferative effect of the root extract was not very high, regular addition of *C. sativum* in the diet could contribute to overall health and wellness and protect against diseases like cancer.

## Conclusion

The ethyl acetate extract of *C. sativum* root has antioxidant and anticancer properties. *C. sativum* root inhibited DNA damage in fibroblasts and prevented MCF-7 breast cancer cell migration induced by H_2_O_2_, suggesting its potential in cancer prevention and inhibition of metastasis. The herb exhibited anticancer activity in MCF-7 breast cancer cells by affecting antioxidant enzymes leading to H_2_O_2_ accumulation, cell cycle arrest at the G_2_/M phase and apoptotic cell death by the death receptor and mitochondrial apoptotic pathways. We report the presence of ascorbic acid in *C. sativum* root, a compound well-known for its antioxidant and anticancer properties. Taken together, we showed that *C. sativum* root has medicinal value with regard to its antioxidant and anticancer properties in preventing oxidative stress-related diseases and may be useful as food or supplements used in combination with conventional drugs to enhance the treatment of diseases such as cancer. The scientific study corroborates the use of this herb in traditional medicine.

## Competing interest

The authors declare that they have no competing interests, financially or otherwise.

## Authors’ contributions

ELHT performed almost all of the experiments and analyzed the data. JR designed the DNA damage study. SYF supervised part of the study and reviewed the manuscript. MSK conceived, designed, analyzed, supervised the study and rewrote the final manuscript. All authors read and approved the final manuscript.

## Pre-publication history

The pre-publication history for this paper can be accessed here:

http://www.biomedcentral.com/1472-6882/13/347/prepub
